# Structural and functional diversity of collectins and ficolins and their relationship to disease

**DOI:** 10.1007/s00281-017-0642-0

**Published:** 2017-09-11

**Authors:** Mark Howard, Conrad A. Farrar, Steven H. Sacks

**Affiliations:** MRC Centre for Transplantation, King’s College London, Guy’s Hospital, 5th Floor Tower Wing, Great Maze Pond, London, SE1 9RT UK

**Keywords:** Lectin pathway, Glycans, Collectin-11, Innate immunity

## Abstract

Pattern recognition molecules are sensors for the innate immune system and trigger a number of pathophysiological functions after interaction with the corresponding ligands on microorganisms or altered mammalian cells. Of those pattern recognition molecules used by the complement system, collagen-like lectins (collectins) are an important subcomponent. Whereas the best known of these collectins, mannose-binding lectin, largely occurs as a circulating protein following production by hepatocytes, the most recently described collectins exhibit strong local biosynthesis. This local production and release of soluble collectin molecules appear to serve local tissue functions at extravascular sites, including a developmental function. In this article, we focus on the characteristics of collectin-11 (CL-11 or CL-K1), whose ubiquitous expression and multiple activities likely reflect a wide biological relevance. Collectin-11 appears to behave as an acute phase protein whose production associated with metabolic and physical stress results in locally targeted inflammation and tissue cell death. Early results indicate the importance of fucosylated ligand marking the injured cells targeted by collectin-11, and we suggest that further characterisation of this and related ligands will lead to better understanding of pathophysiological significance and exploitation for clinical benefit.

## Introduction

This article focuses on the lectin pathway of complement activation, which is the most recent of the complement activation pathways to be described, but the oldest in evolutionary terms [1]. Why now? A number of recent articles have described several new types of collectin, which appear to be distinguished from some of the better-known types of complement activating lectins by their capacity for local tissue production [2–6]. This sets a number of new questions about their participation in local complement function, including organ development and the local response to tissue injury and infection at the portal of entry [7–9]. The purpose of this article is to review what is known about the structure and function of these new lectins and how they differ from other lectins that can trigger complement activation, in respect of their tissue distribution and carbohydrate ligands on different structures. We also consider what, if any, distinctive roles are served by local expression, especially with regard to tissue injury at production sites of other complement components [10]. Lastly and briefly, we speculate about the possibilities for therapeutic and diagnostic application of this knowledge.

## Which pattern recognition receptors are we referring to?

The complement cascade is activated by three major pathways (the classical, lectin and alternative pathways), which lead to C3b deposition on the surface of an invading pathogen. C3 can act as a marker for removal by the mononuclear phagocyte system [11]. Additionally, C3 acts as a marker for the membrane attack complex (MAC), containing C5b and C6–9, which results in the destruction of the pathogen. The pattern recognition receptors (PRRs) of each complement pathway initiate pathway activity via associated serine proteases which leads to sequential cleavage and activation of downstream complement factors, and in so doing they activate the abundant complement pathways intermediate C3 as mentioned above, which represents the convergence point of all three pathways. While the classical pathway has only one PRR, C1q, the lectin pathway has a number of these proteins, including ficolins and collectins (the latter being the focus of this review and discussed below) [12]. The associated serine proteases of the lectin system are the mannose-binding lectin (MBL)-associated serine proteases (MASPs) and they form complexes with the PRRs. Recently, the function of two of these MASPs has been elucidated: MASP-1 is activated first, then in turn activates MASP-2 and then they both participate in the formation of C4b2a convertase (which cleaves C3). A third MASP, MASP-3, has also been described, which despite being able to bind PRRs as other MASPs are, is unable to cleave any parts of the C3 or C5 convertase complexes [reviewed in 13]. An additional important factor of the lectin system and its many PRRs is the location of their synthesis, in that some are synthesised locally and others are synthesised by the liver and are present predominantly in the serum. This means that at any given site, the activation of complement is dependent both on locally produced complement as well as those complement factors that are circulating and/or have diffused into the tissue [11]. This has important implications for the local response to pathogen and altered-self antigen recognition. There follows a description of the main lectin pathway-associated PRRs.

### Ficolins

Ficolins contain both a collagen-like domain and a fibrinogen-like domain that has a specific binding affinity for N-acetylglucosamine. They can act as opsonins and complex with MASPs to activate the complement pathway [14]. Three types of human ficolin are known to date: L-ficolin (ficolin-2), which is synthesised by the liver and is present in the serum [15]; H-ficolin (ficolin-3), which is also synthesised by the liver and the bile duct but additionally in the lung. Lastly M-ficolin (ficolin-1) is synthesised in monocytes, bone marrow, the lung and spleen. All of these types of ficolins oligomerise to form tetramers and hexamers due to interchain disulphide bonds formed by cysteines of the N-terminal region [16]. In mice, only two ficolins have been described, namely ficolin-A and ficolin-B. Ficolin-A is a functional equivalent of ficolin-2 and is present in serum as well as being produced by splenic macrophages and Kuppfer cells. Ficolin-B is a functional equivalent of ficolin-1 and has been described as being present both on cell membranes and in granules of neutrophils and monocytes, but not in the serum [17]. In humans, while ficolin-1 exerts an on-site effect when excreted by monocytes and granulocytes during inflammation and is found at only a low level in serum, both ficolin-2, and to a larger extent, ficolin-3 are found in the serum. Each ficolin recognises a spectrum of ligands and microorganisms through the fibrinogen-like domain. The three ficolins all share a common affinity towards acetylated compounds, although each has different specific binding characteristics [18].

### C-type lectins

C-type lectins (CTLs) are Ca^2+^-dependent lectins that have homologous carbohydrate recognition domains (CRDs) despite displaying binding affinity to a variety of substrates [19]. They comprise 17 different groups that have, during evolution, diversified to interact with a large range of glycan ligands, although some also bind proteins, lipids and inorganic molecules. C-type lectins have a number of functions in vertebrates, including initiation of immune responses, pathogen sensing and homeostasis of serum glycoprotein [20]. The majority of CTL groups outside of collectins (discussed below) do not interact with complement and so will not be discussed here [for more information, see 20, 21].

### Collectins

Collectins are collagen containing CTLs of which nine have been discovered to date: mannose-binding lectin (MBL), surfactant proteins A and D (SP-A and SP-D), collectin-10, also known as collectin liver 1 (CL-10 or CL-L1), collectin placenta 1 (CL-12 or CL-P1), conglutinin, collectins of 43kDA and 46 kDa (CL-43 and CL-46) and collectin-11, also known as collectin kidney 1 (CL-11 or CL-K1) [22]. The alternative nomenclature of a number of these molecules is due to an initial naming based on organ of discovery. Following this, the collectins were renamed to reflect that their expression was not limited to those particular organs (for example, CL-11 (CL-K1) is not restricted to the kidney). The numerical system has been approved by the HUGO gene nomenclature committee and will be used in this review [3]. They contain a collagen-like domain with a short cysteine-rich N-terminus, responsible for the quaternary structure and a CRD responsible for glycan recognition [20]. MBL is the most characterised collectin to date as it was the first to be discovered and has a CRD region that recognises the microbial carbohydrates mannose and N-Acetyl-D-glucosamine (GlcNAc), as well as nucleic acids (in a calcium-dependent manner). In humans, MBL is produced in the liver and is present in the serum as trimers and tetramers [18]. In mouse, there are two MBL genes: *mbl1* and *mbl2*, encoding MBL-A and -C, respectively. They differ in the sizes of their collagen domain and trimerisation domain but retain the same carbohydrate recognition domain [23]. MBL-A and -C are present in the serum as well as being expressed predominantly in the liver, but also in the kidney, brain spleen and muscle [24, 25]. Collectins CL-12, CL-10 and CL-11 are the most recently discovered collectins: CL-10 and CL–11 having classical collectin structure including an N-terminal segment containing cysteine residues followed by a collagen-like region, an alpha helical coiled neck region and a C-terminal CRD [26]. CL-10 and CL-11 circulate in the serum as disulphide bridge-stabilised complexes made up of two CL-11 and one CL-10 subunit [9]. This complex binds to MASPs and has been shown to mediate deposition of C4b in the presence of MASP-2, demonstrating activation of complement [9]. CL-10 has an affinity for d-mannose, N-acetylglucosamine, D-galactose and d-fucose while CL-11 has an affinity for l-fucose, d-mannose and N-acetylmannosamine [26]. The adrenal glands, kidneys and liver are primary sites of CL-11 expression and it is present in the serum [3]. CL-12 by contrast is a single pass (type II) transmembrane protein orientated with the N-terminal facing the cytosol and is expressed in a number of adult tissues, principally the placenta, the lung and the heart [26]. Although collectins have a similarity of structure and many have binding to MASPs in common, they have different specificities in their ligand binding, be it to proteins, glycans or DNA, as well as diverse specificities within those groups.

The PRRs that participate in the complement pathway are numerous and varied, but share a number of similarities as discussed above. It is important to note which PRRs are liver-derived and exist in the serum and which rely on more local synthesis, as this has functional consequences. The PRRs discussed here are summarised in Table 1. Their roles in immunity as well as in disease states will be discussed in the rest of this review.

**Table 1:** Pattern recognition receptors (PRRs) of the lectin system

Pattern recognition receptors (PRRS)			Key observations	Ref(s)
	Nomenclature used in review	Alternate Name		
Ficolins	Ficolin-1 (human)	M-ficolin	Synthesised by monocytes the lung and the spleen and present in the serum, activates complement by complexing with MASPs. Binds to GlcNac residues present in microbe cell wall glycoconjugates and complex oligosaccharides. Homologous to ficolin-B in the mouse, although ficolin-B does not bind MASPs.	[14, 15, 27]
	Ficolin-2 (human)	L-ficolin	Synthesised by the liver and present in the serum. Activates complement by complexing with MASPs. Binds residues as above, in addition to binding DNA. Homologous to ficolin-A in the mouse	[15, 16, 27, 28]
	Ficolin-3 (human)	H-ficolin	Synthesised by the liver and in the lung and present in the serum. Activates complement by complexing with MASPs. Homologue of Ficolin-1 that binds GlcNac and d-fucose, as well as lipopolysaccharide (LPS) on salmonella species, in addition to binding DNA.	[15, 16, 27, 29, 30]
Collectins	Mannose binding Lectin (MBL)		Synthesised by the liver and present in serum as trimers and tetramers. Preferentially binds carbohydrates with 3 and 4 hydroxyl groups of the pyranose ring in a Ca^2+^ dependent manner. Therefore has a higher affinity to mannose and GlcNAc than to galactose and sialic acid. Also binds nucleic acids.	[18, 31–33]
	Surfactant Protein A (SP-A)		Synthesised in the alveolar space of the lung by alveolar type II cells and nonciliated bronchial epithelial cells. Binds calreticulin, CD14, TLR2 amongst other receptors, mediating phagocytosis of microorganisms.	[22, 34–36]
	Surfactant Protein D (SP-D)		Synthesised as above. Binds to CD14 and SIRPα amongst other receptors, mediating the inhibition of cytokine release.	[22, 35, 37, 38]
	Collectin-10 (CL-10)	Collectin liver 1 (CL-L1)	Circulates in the serum as disulphide bridge-stabilised complexes with two CL-11 and one CL-10 subunit. This complex mediates deposition of C4b in the presence of MASP-2. Has a higher affinity for d-mannose, N-acetylglucosamine, D-galactose and d-fucose than other carbohydrate ligands, binding in a Ca^2+^ dependent way.	[6, 9, 26]
	Collectin-11 (CL-11)	Collectin Kidney 1 (CL-K1)	Circulates as above, in addition to being expressed in the adrenal glands, kidneys and liver. Higher affinity for l-fucose, d-mannose and N-acetylmannosamine than other carbohydrate ligands, binding in a Ca^2+^ dependent way. Has both anti-microbial and antifungal roles in host defence.	[3, 6, 26, 39–41]
	Collectin-12 (CL-12)	Collectin placenta 1 (CL-P1)	A transmembrane protein orientated with the N-terminal facing the cytosol. Expressed in a number of adult tissues, chiefly the placenta, the lung and the heart. Binds Lewis^X^, Galactose, T-antigen, Tn-antigen as well as *E.coli* and *S. aureus*.	[26]
	Conglutinin		Only found present in Bovidae, where it is synthesised by the liver. Binds preferentially to GlcNAc and mannosamine.	[22, 42–44]
	Collectin of 43 kDa (CL-43)		Synthesised as above and also present in only Bovidae. Binds preferentially to Mannose and l-fucose.	[22, 42–44]
	Collectin of 46 kDa (CL-46)		Synthesised as above (though also in the thymus) and also present in only Bovidae. Binds preferentially to GlcNAc.	[22, 42, 44]

A summary of the key observations of each of the PRRs in the lectin pathway. This includes current and alternative nomenclature as well as species differences in terminology. These PRRs have contrasting regions of synthesis, binding targets and roles in both immunity and other systems. Further reading is provided for the individual PRRs listed

## What is known about the target glycans on mammalian and microbial structures?

The molecules discussed above function as PRRs of the lectin system, in that they initially recognise pathogen-associated molecular patterns (PAMPs). PAMPS are carbohydrate moieties (particularly hexoses) of glycoproteins or glycolipids on the surface of microorganisms. They also bind damage-associated molecular patterns (DAMPs), namely carbohydrate structures that are displayed on the surfaces of apoptotic, necrotic, malignant, as well as hypoxia and hypothermia-stressed cells. Groups of trimeric CRDs recognise PAMPs on the surface of bacteria and fungi, through multiple weak interactions [45]. Each subgroup of PRRs binds in unique ways to different target molecules.

### Mannose-binding lectin

Structural analysis of mannose-binding lectin (MBL) complexed with an oligomannose asparaginyl-oligosaccharide showed that Ca^2+^ forms coordination bonds with the carbohydrate ligand and specificity is stabilised by interaction of protein (MBL), Ca^2+^ and oligosaccharide [31]. This study also determined that the CRD of MBL has a preference for binding carbohydrates with 3- and 4-hydroxyl groups of the pyranose ring, with Ca^2+^ required for this [31]. This steric positioning enables MBL to bind with higher affinity to ligands such as mannose and GlcNAc than carbohydrates such as galactose and sialic acid [32]. The immune response to the trematode *Schistosoma haematobium* requires activation of the LP by MBL, which binds to schistosomal glycoconjugates [46]. MBL is known to mediate clearance of apoptotic debris through binding of nucleic acids displayed on the surface of apoptotic cells [47]. MBL binds nucleic acids via its CRD in a Ca^2+^-dependent manner and binds more avidly to dsDNA than ssDNA or ssRNA [33]. It is postulated that binding of nucleic acid displayed on apoptotic cell surfaces promotes phagocytosis, thereby curtailing autoimmunity and helping to maintain tissue homeostasis.

### Collectin (CL-11 and CL-10)

Ca^2+^ binding is also important for CL-11 secretion, as mutations in the Ca^2+^ binding region prevent extracellular release of CL-11 [48]. This study also demonstrated that CL-11 bound to subsets of fucosylated glycans, such as blood group B antigens and fucosylated glycans containing Lewis^a^ and Lewis^y^ [48]. Structures containing blood group H antigens were also bound by CL-11. Calcium is an essential component for the binding of collectin to the carbohydrate as it maintains the tertiary structure of the carbohydrate recognition domain. CL-11 binds with higher affinity to l-fucose, d-mannose and α-methyl-d-mannose than for *N*-Acetyl-d-mannosamine (ManNAc) and d-glucose and still lower affinity for d-galactose of all other monosaccharides tested [3]. The authors concluded that CL-11 binds mannose-related hexoses or l-fucose-related pentoses that are prevalent in microbial glycoconjugates. It has been shown that CL-11 binds strongly to l-fucose but has relatively weak affinity for GlcNAc and early analyses determined that CL-11 is widely distributed in humans [4], with prominent expression at the protein level in mice in the adrenal glands, liver, kidney, small intestine and embryonic tissue [49]. Indeed, in the kidney, it was recently demonstrated that complement-mediated parenchymal damage is triggered by activation of the LP, in an l-fucose-dependent manner, raising the likelihood that fucosylated endogenous ligands are engaged by CL-11 (mentioned in more detail later) [7]. CL-11 and CL-10 have also been shown to form hetero-oligomers, which make up a significant proportion of all circulating CL-11 and CL-10 [9], and direct complement activation in a MASP-2-dependent fashion. Native CL-10 present in serum is known to exhibit a different binding profile to the recombinant form of the protein, with rCL-10 binding to mannose, fucose and galactose [5]. In serum, CL-10 binds to mannose, binding that was inhibited by l-fucose, GlcNAc and ManNAc but not by galactose, suggesting that galactose is not a ligand for this collectin [2]. However, these variations in carbohydrate recognition between recombinant and serum forms may reflect differential binding capabilities of the heteromeric form of CL-10 with CL-11 described above. A recent study highlighted the diversity of CL-11 roles conferred by its differential ligand specificity: in a similar manner to MBL but in a Ca^2+^-independent manner, CL-11 plays a role in the clearance of apoptotic cells through engagement of DNA displayed on their surface [50]. CL-11 has anti-microbial activity in that it has been shown to bind *Streptococcus pneumoniae*, driving LP activation as an immune defence mechanism against infection [39, 40]. CL-11 has the capability to bind LPS from a number of *E. coli* strains and *Klebsiella pneumoniae* and *Salmonella minnesota*, as well as lipoteichoic acid (LTA) from *Streptococcus pyogenes* [4]. CL-11 is important in host defence against the fungus *C. albicans*, the mechanism of which is postulated to be via CRD domain recognition of CL-11, as binding to *C. albicans* was disrupted with mannose, GlcNAc or chelation of Ca^2+^ [41].

### Ficolins

Ficolins have collagen-like and fibrinogen-like domains and are able to activate complement via the LP [1]. A unique feature of ficolin molecules is that carbohydrate recognition is mediated via the fibrinogen-like domain. They have prominent binding affinity to GlcNac residues present in microbe cell wall glycoconjugates and complex oligosaccharides [27]. This was also elegantly demonstrated in an infection model in which binding of recombinant ficolin proteins to the D39 strain of *S. pneumoniae* was inhibited by GlcNAc [51]. Ficolin-3 (originally termed Hakata antigen) is a homologue of ficolin-1 and is known to bind to GlcNac and d-fucose, whilst also having affinity for lipopolysaccharide (LPS) on salmonella species [29]. With functional similarity to both MBL and CL-11, ficolins-2 and -3 have the ability to bind DNA, consistent with a role in the clearance of apoptotic cell debris by phagocytic cells to prevent autoimmunity [28, 30]. There are a number of other varying microbial and viral binding specificities of the ficolin molecules that are comprehensively discussed in a recent review [18]. In addition, glycan array screening has shown the variety of these molecules in terms of their binding targets [52]. For ficolin-3, no significant binding was found to any of the glycans tested in this study which suggests a role for binding to non-carbohydrate acetylated ligands. It is important to note that the database used for the glycan array is based on mammalian carbohydrates so does not exclude those specific to pathogens [more information in 53]. Ficolins-1 and -2 show differing glycan binding profiles, ficolin-1 binds to sialic acid and its derivatives while ficolin-2 binds to saccharides with Gal or GlcNAc residues [18, 52].

## Where do lectin pathway components appear in evolution and what does this tell us?

The phylogenetic relationship between the CRD amino acid sequences has been determined in the MBL group of collectins, the surfactant collectins (SP-A and SP-D groups) and those of CL-11, CL-10 and CL-12 [6]. From their genomic organisation, lectin activity and expression profiles, the suggestion is that CL-11 and CL-10 evolved similarly [4]. Analyses of chicken homologues of CL-10, CL-11 and CL-P1 revealed that this group of collectins represent a new unique class in the collectin family. The CRDs of chicken collectins (termed CL-1, CL-2 and CL-3) and those of their mammalian homologues CL-10, CL-11 and CL-12 are found in well-supported clades, demonstrating that these proteins share a common ancestor [54]. Genome analysis of early invertebrate life forms such as sea urchins showed that they utilise primitive collectins, which constitute an evolutionarily ancient form of the innate immune system [1]; these evolved into a larger number of collectin genes in amphioxus, a species that have cartilage-like material but no true skeleton [55]. The evolution of collectin genes has occurred during more than one distinct phase. Firstly, when amphioxus evolved from the sea urchin, amphioxus retained a total of 66 collectin genes. In subsequent evolutionary steps, when urochordates evolved into fish, just four collectin genes were retained. Of particular interest is the observation that zebrafish express CL-11 but do not have the gene for CL-10 [6]. It is during the evolutionary transition from invertebrate to vertebrates that CL-11, CL-10 and CL-12 appear. This raises the question of a succinct role for this family of collectins in the orchestration of skeletal developmental processes. Indeed, this connection has recently been observed [8, 56].

## What is the wider biological significance of these molecules in medicine?

The PRRs detailed in this review have important roles in a number of different biological processes, from immune functions such as antimicrobial defence and inflammation to separate processes such as development.

Through MASPs, the LP also has a role in the coagulation pathway [57]. Following a vascular injury, the coagulation pathway is activated at the site of injury (rather than that of infection) and results in the generation of a blood clot [58]. MASP-1 resembles thrombin in its activity in that it cleaves factor VIII, fibrinogen and thrombin activatable fibrinolysis inhibitor (TAFI). MASP-2 by contrast cleaves solely prothrombin [59]. Complexes of both MBL-MASPs and ficolin-2-MASPs bound to glycans generate clots similar to those generated by thrombin when supplied with fibrinogen and factor XIII [60]. This demonstrates that the activated PRRs of the LP bound to their serine protease can crosstalk with and activate the coagulation pathway. These clots also then have the potential to surround infectious microbes and by so doing slow their spread [57].

In contrast to the biological process in which lectin-glycan binding is significant (discussed above), there is increasing evidence that these interactions are relevant in development. Firstly, MASP-3 selectively cleaves insulin-like growth factor binding protein 5 (IGFBP5). IGFBP5 binds to insulin growth factors (IGFs) in the extracellular space and in turn influences their interactions with the cell surface, but the cleavage of IGFBP5 blocks this binding [61]. IGFs have a range of function including modulation of cell differentiation, proliferation, survival and motility, all key cellular aspects in development. In addition to this, MASP-1 has been shown to activate human endothelial cells through protease activated receptor 4 (PAR4) [62]. PAR4 is also a mediator of platelet activation and an important factor in inflammation [57]. In the same study, it was also shown that MASP-1 is able to signal through the Ca^2+^ signalling pathway, p38 MAPK pathway and the NF-κβ pathway in these human endothelial cells [62]. The range of signalling pathways and target cell types through which it has been shown that LP proteins can signal provide evidence for further roles in many key biological areas including development.

**Fig. 1:**
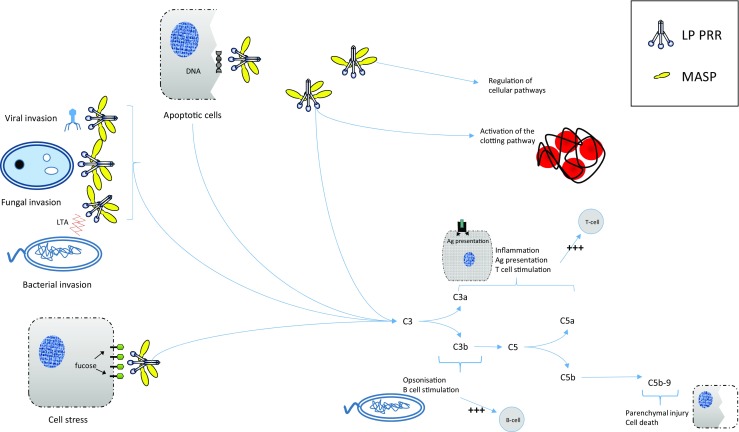
Roles of lectin pathway PRRs in multiple situations and important processes. Upon presentation of a number of different situations; cell stress, bacterial, viral or fungal invasion and apoptotic cells, the PRRs of the lectin pathway bind to substrates and through MASPs cause lectin pathway mediated cleavage of C3. This stimulates inflammation, enhances antigen presentation and T and B cell responses, opsonisation, parenchymal injury and cell death. In addition, PRRs of the lectin pathway have roles in the clotting cascade and MASPs themselves can aid in the regulation of certain cellular and developmental pathways. *PRR* pattern recognition receptor, *LP* lectin pathway, *MASP* MBL-associated serine protease, *Ag* antigen, *LTA* lipoteichoic acid. Note that the binding ligand for CL-11 on mammalian cells is represented as a monosaccharide, when in reality it forms part of complex polysaccharides

Evidence for a specific role of LP in development has surfaced through study of a rare human syndrome. Malpuech-Michels-Mingarelli-Carnevale (3MC) syndrome is characterised by a range of developmental defects, including cranial abnormalities, cleft lip and palate, limb anomalies and learning deficiencies. Studies of families with this syndrome have identified mutations in genes of the lectin pathway, namely CL-11 and its associated serine protease MASP-1 [8]. Utilising morpholino studies in zebrafish, the authors confirmed that disruption of CL-11 or MASP-3 gene expression caused striking craniofacial abnormalities, an effect that was additive when both genes were disrupted. Further investigation determined that CL-11 behaves as a chemoattractant and directs the migration of neural crest cells for development. A more recent study described mutations of the CL-10 gene in 3MC syndrome patients, again linking LP activity to morphogenesis [56]. This hitherto unknown function of collectins in embryogenesis and development is illuminating, and it would be of importance to consider the possibility that cell-cell interactions during development may involve glycan ligand recognition by CL-11 in conjunction with the proposed role of CL-11 as a chemoattractant. These processes and the activating factors are summarised in Fig. 1.

## What is the evidence that glycan-lectin interaction contributes to complement-mediated disease?

Aberrant regulation of complement has a role in diverse diseases, including Alzheimer’s disease, macular degeneration, diabetes and epilepsy, amongst many others. The diversity of these diseases belies a common pathway that is typically followed. Beginning with the (often false) recognition of danger, a dysregulation of various amplification processes follows, with the result that a downstream inflammatory response is activated. Other parts of the immune system can exacerbate this process, as well as increasing levels of tissue damage. In addition to the damage, the involvement of other immune pathways can result in further danger signals causing the formation of an endless loop of further activating complement pathways [reviewed in more detail in 63]. The aforementioned complement-mediated diseases show the consequences of this type of inappropriate complement activation control and/or deposition [reviewed in 64]. The interactions between glycans and lectins specifically result in numerous instances where complement is inappropriately triggered. For example, in the field of organ transplantation, the role of complement has become increasingly recognised [11]. Peripheral synthesis of complement is important in the fate of tissue undergoing the stress of the surgical procedure from organ donation as well as ischaemia-reperfusion injury. This synthesis also contributes to T cell priming and transplant rejection through adaptive immune responses [65]. In addition, MASP-2 levels have been linked to several diseases including schizophrenia, acute lymphoblastic leukaemia, non-Hodgkin lymphoma and colorectal cancer amongst others, suggesting a role for MASP-2 (downstream of lectin-glycan interactions) in human disease [66]. Furthermore, complement has a role in some non-infectious diseases where the lectin-glycan interactions are no longer appropriate and a few selected examples of these will be discussed below.

The collagen antibody-induced arthritis (CAIA) mouse model involves the formation of immune complexes of anti-collagen type II antibodies and collagen type II within the joint. Collagen type II is a major component of the articular cartilage matrix. These immune complexes model the progression of rheumatoid arthritis (RA), where autoantibodies, especially those in immune complexes, play a key role in the promotion of inflammation in the disease [67]. Although previous work had suggested that the alternative pathway was the only pathway necessary for this model, as C4 [68], C1q and MBL knockout mice [69] show no protection. An endogenous inhibitor of all MASPs, namely MAp44, was used to interrogate fully the role of the LP in this model. MAp44 is a product of alternative splicing at the MASP-1/3 gene that competes with the binding of recognition molecules such as MBL, ficolins and collectins to all three MASPs. The addition of this inhibitor prevented the initiation of arthritis in the CAIA model mice, which was accompanied by a decrease in cartilage and synovial C3b deposition (demonstrating that the effect was complement-dependent), as well as improvements in histology scores [67]. Interestingly, this ameliorating effect was also present when MAp44 was tested in both myocardial ischaemia/reperfusion (I/R) injury and thrombogenesis models [70]. In human populations, an SNP in the ficolin-1 gene (FCN-1) is also associated with RA [71]. The demonstration that downstream inhibition of the lectin pathway using MAp44 is protective for models of RA, myocardial I/R injury and thrombosis, suggests a role for the lectin pathway in all of these pathologies. It is a distinct possibility that glycans displayed as a result of the initial insult, through either immune complex formation in the CAIA model or hypoxia-induced damage in the I/R injury model, lead to this aberrant lectin pathway-mediated damage.

In addition to the effect of the LP on myocardial I/R injury, other causes of heart disease have been linked to LP activation. Indeed, MBL-2 polymorphisms predispose to myocardial infarction [72]. MASP-2 has been shown to be reduced in the peripheral blood of myocardial infarct patients as well as those in a coronary artery bypass grafting setting and MASP-2 levels correlated with an increase in a myocardial necrosis marker [73]. Together, this suggests that genetic reductions in MBL reduce the activation of the LP in disease states, though this may not be through MASP-linked processes, as the effects of MASPs seem more variable [74]. Interestingly, plasma levels of MAC are also associated with unstable coronary disease [75], suggesting that there are other mechanisms through which lectins and complement are involved in cardiac pathologies [74].

Finally, in a renal I/R model, lectins have been shown to be an important mediator of tissue damage. I/R is a unavoidable consequence of transplantation and approximately one third of organ transplants receive significant I/R damage (increasing to one half when the donor has circulatory arrest) [11]. This damage is the cause of delayed graft function which both accelerates acute rejection and reduces the long-term graft survival [76]. The impaired oxygen and nutrient delivery as well as reduced waste removal are associated with the tubular epithelial cells becoming injured, which can lead to apoptosis in severe cases [reviewed in 77]. Significantly, this causes a change in the morphology of cells, including the molecules presented on the cell surface. It has been demonstrated a number of times that MASP-2 is essential for this injury [76, 78], suggesting the involvement of the LP [79] and there is strong evidence that lectin-glycan binding is responsible for the activation of the LP in this process.

A mouse model of this process applied to a CL-11 knockout mouse demonstrated the relevance of glycan-lectin binding in the activation of complement in response to I/R injury. The CL-11 knockout mice demonstrated a reduced level of tissue injury following renal I/R. Furthermore, in kidney tubule cell cultures prepared from WT mice, hypoxia caused an increase in CL-11 expression and release, with prominent deposition on the basal cell surface. Investigations in renal tubule cells from the knockout mice demonstrated that these cells could bind exogenously added CL-11 in the presence of cell stress [7]. l-fucose has emerged as a key ligand of CL-11 when it binds to bacteria [3]. l-fucose is a deoxyhexose which forms part of several glycans that have roles in several processes, including blood transfusion reactions, host-microbe interactions and many ontogenic events. It can be presented on the cell membrane of both host cells and bacteria [reviewed in 80]. In CL-11 knockout renal tubule cells, pre-treatment of CL-11 with l-fucose prevented its binding to the cell surface, an effect that was not observed upon use of D-galactose. Further to this, once l-fucose is enzymatically removed from the surface of these cells, they showed a reduced ability to bind CL-11 [7]. This combined evidence suggests that l-fucose presentation on the surface of renal tubule cells is critical for the binding of CL-11 and that this interaction triggers the LP complement cascade. This observation raises the possibility that l-fucose could be applied as a therapeutic agent in a wider setting, to prevent complement-mediated I/R injury, which will be further discussed below.

## What conclusions can we draw and how could this be taken forward?

Tissue-based PRRs offer numerous new challenges and opportunities due to the diversity and unique features of some of the functions that have been identified to date. It remains to be shown whether this diversity, ranging from developmental activity to immune recognition and tissue injury can be integrated or at least understood by the precise nature of the ligand-collectin interaction, and how these interactions may differ at different stages of development or in the context of pathophysiological stress in the fully formed organ. It also remains to be determined whether there could be cross-reactivity between the structures recognised on pathogens and those on stressed tissue cells, and whether this could shed light on theories of molecular mimicry in some inflammatory conditions. It would seem that a more precise determination of the nature of the glycan ligands and their interaction with tissue bound collectins could hold the key to fundamental advance.

Better understanding of the major ligands recognised by these lectins could also lead to development of new therapeutics relevant to clinical application. For example, we have generated early data showing that soluble l-fucose can act as a decoy molecule, obstructing the carbohydrate recognition site on CL-11 and preventing tissue injury following the induction of I/R in mice (unpublished data). An alternative approach to blocking this interaction would be the use of glycosylation pathway inhibitors that prevent the expression of fucosylated glycoproteins or glycolipids on the cell surface. This approach is being advanced in cancer therapy to block tumour metastasis. Of note, orally active inhibitors of fucosylation are showing potential in preventing tumour engraftment and outgrowth [81]. The use of MASP-2 inhibitors is also a reality, since MASP-2 is required for lectin pathway activation of complement initiated by several PRRs and complete inhibition of the lectin pathway is observed in the absence of MASP-2 [82]. Accordingly, use of anti-MASP-2 as a therapeutic agent has generated considerable interest. Indeed, therapeutic use of anti-MASP-2 antibody has demonstrable efficacy in a variety of native ischaemia models [83, 84] and Omeros OMS271, a commercial anti-MASP-2, is currently in phase 2 clinical trial for treatment of thrombotic microangiopathies (TMAs) including atypical hemolytic urea syndrome (aHUS) [85].

With regard to biomarker development, there is early evidence that CL-11 levels in peripheral blood may be associated with renal transplant outcome [86]. Genetic variants in the *FCN2* gene double the chance of bacterial infection in the first year following liver transplant, a risk further increased with the coinheritance of *MBL2* gene variants [87]. And, as already mentioned, the presence of elevated levels of CL-11 in peripheral blood has been linked with a thrombotic tendency in acutely ill patients. There is a case to evaluate urinary or plasma measurement of CL-11 in different types of acute and chronic kidney conditions, to assess the sensitivity and specificity for early diagnosis and monitoring of renal tubule injury, against other established markers such as kidney injury molecule-1 (KIM-1) [88]. In addition, ficolin-2 levels have been linked to prematurity, low birth weight and infections in neonates. The same study found that LP activity correlated with low birthweight but MBL levels did not [89]. These wide ranging findings suggest that PRRs of the lectin system are a viable avenue for future research into biomarkers for a number of different diseases.
